# Clinical Outcomes After Ventriculo-Peritoneal Shunting in Patients With Classic vs. Complex NPH

**DOI:** 10.3389/fneur.2022.868000

**Published:** 2022-07-12

**Authors:** Eng Tah Goh, Christine Lock, Audrey Jia Luan Tan, Bee Ling Tan, Sai Liang, Robin Pillay, Sumeet Kumar, Azlina Ahmad-Annuar, Vairavan Narayanan, Janell Kwok, Yi Jayne Tan, Adeline SL Ng, Eng King Tan, Zofia Czosnyka, Marek Czosnyka, John D. Pickard, Nicole C. Keong

**Affiliations:** ^1^Department of Neurosurgery, National Neuroscience Institute, Singapore, Singapore; ^2^Department of Neuroradiology, National Neuroscience Institute, Singapore, Singapore; ^3^Department of Biomedical Science, Faculty of Medicine, University of Malaya, Kuala Lumpur, Malaysia; ^4^Division of Neurosurgery, Department of Surgery, Faculty of Medicine, University of Malaya, Kuala Lumpur, Malaysia; ^5^Department of Neurology, National Neuroscience Institute, Singapore, Singapore; ^6^Duke-NUS Medical School, Singapore, Singapore; ^7^Neurosurgical Division, Department of Clinical Neurosciences, University of Cambridge, Cambridge, United Kingdom

**Keywords:** Alzheimer's disease, dementia, neurodegenerative disease, Normal Pressure Hydrocephalus (NPH), Parkinson's disease, ventriculo-peritoneal shunt (VPS)

## Abstract

**Objective:**

Normal pressure hydrocephalus (NPH) is a neurological condition characterized by a clinical triad of gait disturbance, cognitive impairment, and urinary incontinence in conjunction with ventriculomegaly. Other neurodegenerative diseases, such as Alzheimer's disease, Parkinson's disease, and vascular dementia share some overlapping clinical features. However, there is evidence that patients with comorbid NPH and Alzheimer's or Parkinson's disease may still exhibit good clinical response after CSF diversion. This study aims to evaluate clinical responses after ventriculo-peritoneal shunt (VPS) in a cohort of patients with coexisting NPH and neurodegenerative disease.

**Methods:**

The study has two components; (i) a pilot study was performed that specifically focused upon patients with Complex NPH and following the inclusion of the Complex NPH subtype into consideration for the clinical NPH programme, (ii) a retrospective snapshot study was performed to confirm and characterize differences between Classic and Complex NPH patients being seen consecutively over the course of 1 year within a working subspecialist NPH clinic. We studied the characteristics of patients with Complex NPH, utilizing clinical risk stratification and multimodal biomarkers.

**Results:**

There was no significant difference between responders and non-responders to CSF diversion on comorbidity scales. After VPS insertion, significantly more Classic NPH patients had improved cognition compared to Complex NPH patients (*p* = 0.005). Improvement in gait and urinary symptoms did not differ between the groups. 26% of the Classic NPH group showed global improvement of the triad, and 42% improved in two domains. Although only 8% showed global improvement of the triad, all Complex NPH patients improved in gait.

**Conclusions:**

Our study has demonstrated that the presence of neurodegenerative disorders co-existing with NPH should not be the sole barrier to the consideration of high-volume tap test or lumbar drainage *via* a specialist NPH programme. Further characterization of distinct cohorts of NPH with differing degrees of CSF responsiveness due to overlay from neurodegenerative or comorbidity risk burden may aid toward more precise prognostication and treatment strategies. We propose a simplistic conceptual framework to describe NPH by its Classic vs. Complex subtypes to promote the clinical paradigm shift toward subspecialist geriatric neurosurgery by addressing needs for rapid screening tools at the clinical-research interface.

## Introduction

The pathophysiology of Normal Pressure Hydrocephalus (NPH) still provokes great debate. It is classically a characterized by a clinical triad of gait disturbance, cognitive impairment and urinary incontinence in conjunction with radiographic findings of ventriculomegaly (1). Other neurodegenerative diseases, such as Alzheimer's disease, Parkinson's disease or parkinsonism, and vascular dementia share some overlapping clinical features. Certain phenotypes of disease, namely NPH with comorbidities, are especially difficult to treat. Patients who present with neurodegenerative disease or vascular risk burden are considered poor candidates for intervention. Studies have postulated that such patients may have dual-pathology. Yet, their clinical characteristics are also atypical for neurodegeneration, such as Alzheimer's or Parkinson's diseases. Brain biopsy or advanced imaging such as single-photon emission computed tomography (SPECT) scans may provide for accurate diagnosis to aid the prediction of surgical outcomes but are either highly invasive or impracticable to deliver in screening programmes for NPH. This clinical conundrum results in such patients receiving neither intervention for neurodegenerative disease, nor consideration of CSF shunting.

The co-existence of NPH with neurodegenerative diseases has been reported (2–6), and several studies have described good clinical response after CSF diversion in patients with NPH, coexisting with Parkinsonism or Alzheimer's disease (7–11). It is known that such cohorts may demonstrate a degree of responsiveness to CSF drainage. Methodical supplementary testing and risk stratification are critical to understanding the potential scope for intervention. Identification of comorbidities is an important aspect of the management of idiopathic NPH (INPH). Indeed, this was the basis of a consensus statement by the International Society for Hydrocephalus and Cerebrospinal Fluid Disorders (ISHCSF) (12). However, the relative contribution of comorbidities to CSF responsiveness has not been examined even in large studies on INPH. Few studies have focused on NPH patients with significant comorbidities as a distinct cohort presenting within the spectrum of INPH. The cohort of NPH patients with comorbidities are believed to have a higher risk of non-responsiveness to CSF drainage than typical INPH patients (i.e., the classic cohort of NPH). Yet, the influence of individual comorbidities upon CSF responsiveness is uncertain.

In this study, we present the results of a progressive multi-year change in practice within a subspecialist NPH programme, to understand and adapt to the needs of this uncertainty. Firstly, we performed a pilot study focused primarily on characterizing the risk stratification of patients presenting with NPH but with overlay from significant vascular and neurodegenerative disease. As these patients demonstrate features of Classic NPH but both their level of CSF responsiveness and degree of comorbidities need to be quantified, we have proposed the term “Complex NPH” to distinguish this cohort from more typical patients within the INPH spectrum. Key features of this pilot include (i) the use of global risk scoring and multi-modal MRI for the characterization of Complex NPH (ii), correlation of semi-automated quantitative MRI changes with responsiveness to CSF drainage and (iii) genotyping for assessment of neurodegenerative risk potential. Our aim was to produce a well-characterized dataset of biomarkers that may serve as a basis for the future evaluation and consideration of such patients as a separate cohort of complex NPH as distinct from Classic NPH, in which more standard levels of responsiveness to CSF diversion would be expected. Following work in the pilot, we then performed a retrospective study on 1 year of consecutive patients with NPH being seen via a subspecialist clinic. We examined this unfiltered patient group for subtypes of NPH presentation, shunting intervention in cohorts of Classic vs. Complex NPH and confirmed their outcomes at 2 years.

## Materials and Methods

### Study Setting and Rationale

**Figure 1:**
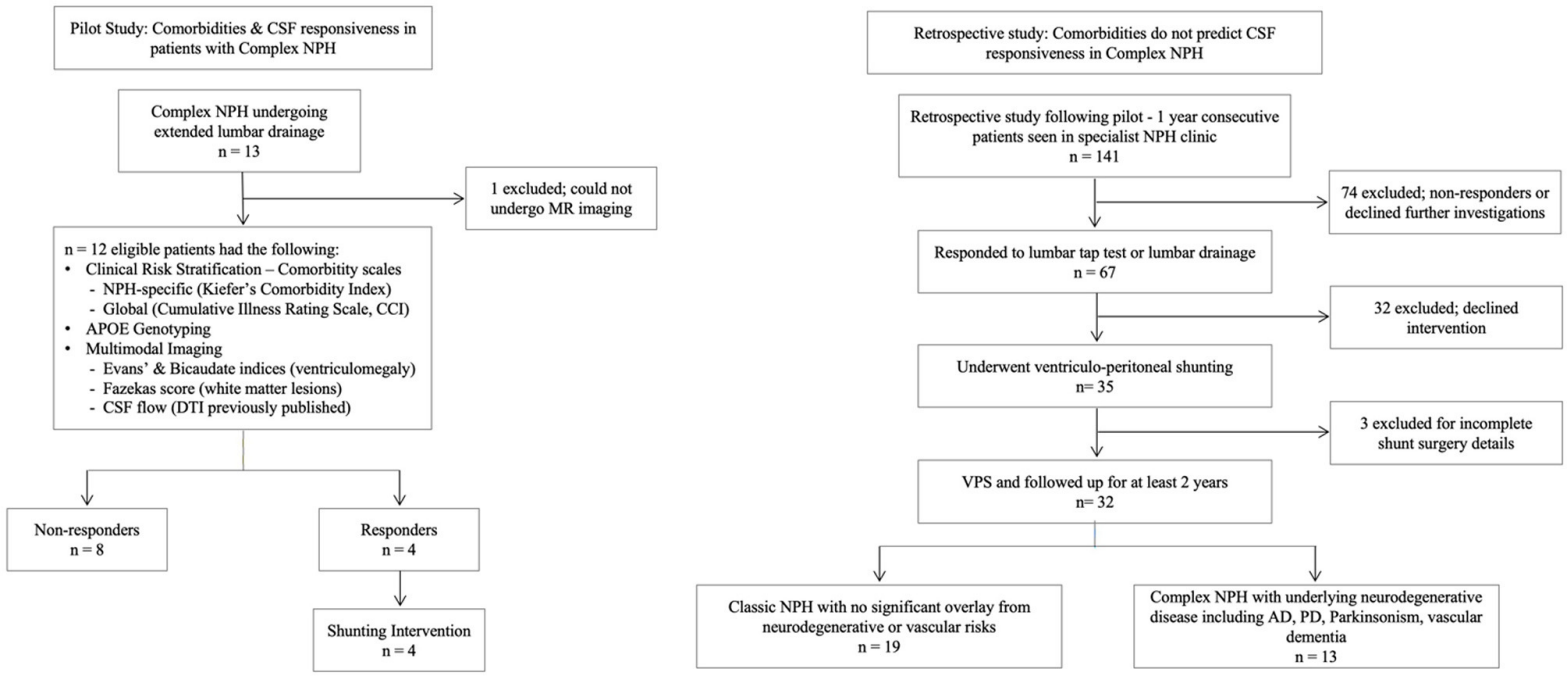
Flow diagram describing the pilot and restrospective studies.

The study has two components; (i) a pilot study was performed that specifically focused upon patients with Complex NPH and following the inclusion of the Complex NPH subtype into consideration for the clinical NPH programme, (ii) a retrospective snapshot study was performed to confirm and characterize differences between Classic and Complex NPH patients being seen consecutively over the course of 1 year within a working subspecialist NPH clinic. In the pilot study, we studied the characteristics of patients with Complex NPH, utilizing clinical risk stratification and multimodal biomarkers (including imaging biomarkers and genotypic risk). We had previously published the characteristics of white matter injury in this cohort using diffusion tensor imaging (DTI) (13). We found that comorbidities did not predict CSF responsiveness in our cohort of Complex NPH. In the retrospective study, we performed clinical risk stratification according to our revised conceptual framework of Classic vs. Complex NPH instead. The two study components and cohorts are illustrated in Figure 1.

### Study Samples

In the pilot study, 13 patients diagnosed with Complex NPH undergoing the extended CSF drainage protocol under the NPH programme at the National Neuroscience Institute, Singapore between 2016 and 2017 were selected for the study. We recruited NPH patients with known significant comorbidities, such as cardiovascular risk burden or neurodegenerative disease overlay from all patients presenting with NPH and meeting clinical criteria for further testing. Within the NPH programme, patients who were deemed to have shown significantly positive responses to high-volume tap testing were offered shunt insertion without additional supplementary testing (13). However, patients who demonstrated low or borderline positive results on tap testing or had comorbidities confounding the assessment of short-term responsiveness to CSF drainage were offered the extended CSF drainage protocol. We have previously published the DTI profiles and clinical characteristics for this pilot study cohort of patients with Complex NPH (13). In this study, we present multi-modal MR findings and biomarkers to elucidate the characteristics of Complex NPH not previously reported in our prior work.

In the retrospective study, we screened the list of outpatients under a single surgeon (NCK) in at the National Neuroscience Institute from October 2017 to October 2018. 141 patients were reviewed in out-patient clinic for probable NPH, idiopathic NPH and secondary NPH. 67 out of these 141 patients were responders who demonstrated a positive response to a lumbar tap test or lumbar drainage. Only 35 responders underwent ventriculo-peritoneal shunt (VPS) insertion. Among these 35 patients, 2 patients who had VPS insertion more than 10 years ago and 1 patient who had surgery in a private hospital were excluded.

### Diagnosis of NPH

Clinical descriptions for all patients met criteria, according to published guidelines, for either probable or possible NPH (14). Patients referred to the NPH programme but deemed unlikely to have NPH were excluded from further testing. All patients met the criteria for communicating hydrocephalus. We have previously published our criteria for defining ventriculomegaly as an Evans' index (maximum width of frontal horns of the lateral ventricles divided by the transverse inner diameter of the skull) ≥0.30, and a Bicaudate index (minimum intercaudate distance divided by the brain width along the same line) ≥0.25 (13).

In our study, patients with probable NPH demonstrated two or more features of the classic clinical triad of gait disturbance, cognitive impairment and urinary incontinence. Where patients were deemed to have possible NPH, they exhibited symptoms of either (a) incontinence and/or cognitive impairment, but without significant gait disturbance or (b) gait dysfunction or cognitive impairment alone. As per guidelines, these symptoms in patients with probable or possible NPH were not thought likely to be entirely attributable to other neurological, psychiatric, or general medical disorders, even if such disorders were found to be co-existing (14). All patients referred to the NPH programme were independently evaluated by subspecialist neurology or geriatric teams and did not solely fit the diagnostic criteria for Alzheimer's and Parkinson's diseases, and/or had limited response to disease-modifying drugs such as levodopa. Patients were only offered entry into the NPH programme, including supplementary testing, if they were not of more than moderate risk of surgical intervention.

### Methodologies for the Pilot vs. Retrospective Study Components

In the pilot, all participants underwent insertion of a lumbar drain to according to our previously published extended CSF drainage protocol (13). Two participants required the insertion of an Ommaya reservoir for testing due to failure to achieve lumbar drainage. One patient exhibited significant psycho-behavioral symptoms and could not cooperate with MR imaging. Whilst he did have ventriculo-peritoneal shunting, we excluded him from subsequent study analysis. 12 participants underwent the full NPH programme for CSF drainage according to our previously published protocol (13) including clinical gait and cognitive testing, pre- and post-drainage inpatient MR imaging and *APOE* genomic analysis. Patients with a lumbar drain *in-situ* underwent a three-day drainage/ seven-day global assessment protocol, achieving ≥300 mls total CSF withdrawal whereas the patient undergoing serial taps *via* Ommaya reservoir had a modified protocol achieving ≥150–200 mls total CSF withdrawal to account for the tolerance needed for more rapid drainage and increased infection risk from repeated tapping.

In the retrospective study component, a final set of 32 NPH patients was included in this study. Demographic and clinical data, including age, sex, comorbidities, presenting symptoms and clinical responses after VPS were collected retrospectively from clinical records. Tinetti gait and balance scores pre- and post-VPS insertion were collected (1 Complex NPH patient did not have pre- and post-VPS Tinetti). Mini-Mental State Examination (MMSE) scores pre-VPS are reported in this study (2 Classic NPH patients did not have pre-VPS MMSE), although post-VPS MMSE scores were excluded from analysis due to missing data.

We categorized these patients into the proposed conceptual framework; (i) Complex NPH, comprising 13 patients had underlying neurodegenerative diseases which include Alzheimer's disease, Parkinson's disease or parkinsonism, and vascular dementia, and (ii) Classic NPH, the other 19 patients who did not have such clinical risk burden. The diagnoses of neurodegenerative diseases were established by either a neurologist or geriatrician prior to referral to the NPH programme under the Department of Neurosurgery.

### Comorbidity and Frailty Risk Assessments

Three comorbidity scales were used to risk stratify the pilot study cohort—Kiefer's Comorbidity Index, the Cumulative Illness Rating Scale, and the Charlson Comorbidity Index; each was scored by two independent raters. Kiefer's Comorbidity Index (CMI) is an NPH-specific scale, with patients scoring >3 points less likely to be shunt-responsive (15–17). We further refined CMI grading to clarify the cerebral infarction criterion as meaning lobar or territorial strokes, excluding lacunar infarcts and microhaemorrhages. We used the latter two scales to report global comorbidity risk burden.

In scoring the Modified Cumulative Illness Rating Scale (CIRS), we used guidelines adapted by Salvi et al. (18) for elderly patients with more complex comorbidities but modified it for use in NPH. We considered ventriculomegaly or NPH diagnosis as primary conditions; these were therefore excluded as neurological comorbidities for scoring, to demonstrate overlay from other neurological diagnoses. Similarly, we excluded age as a comorbidity on the Charlson Comorbidity Index (CCI) (19), since NPH is almost exclusively seen in the elderly population.

In the retrospective study, we explored the use of frailty indices to describe NPH cohorts. Frailty has been shown to be predictive of health outcomes and post-operative morbidity and mortality (20–22). Frailty risk was scored using the Canadian Study of Health and Aging Clinical Frailty Scale (CFS) and the 11-factor modified frailty index (mFI−11).

### Imaging

MR imaging data for the pilot study were acquired with a 3.0-T MR scanner (Ingenia, Philips Medical Systems, Best, the Netherlands), including 3D T1, T2 and FLAIR sequences. A few patients were downgraded to the 1.5-T scanner due to clinical MR safety concerns.

Fazekas scoring for white matter intensities was performed for all pre- and post-lumbar drainage FLAIR MRI sequences and independently verified by a second rater. We rated the periventricular white matter and deep white matter scores based on the presence, size and confluence of white matter lesions (23). Scoring was performed according to established convention; periventricular white matter ratings of 0 = absent, 1 = pencil-thin lining, 2 = “halo”, 3 = irregular periventricular signal extending into deep white matter and deep white matter ratings of 0 = absent, 1 = punctate foci, 2 = beginning of confluence, 3 = large, gathered confluence. Confluence of white matter lesions were considered present if seen across two or more imaging cuts with extensions into deep white matter.

### Genomic Analysis

Genomic DNA was obtained from leukocytes using the QIAamp® DNA Blood Midi kit (Qiagen GmbH, Hilden, Germany). *APOE* genotypes were determined using Taqman® allelic discrimination assays (for SNPs rs7412 and rs429358) and genotyping was carried out on a 7,500 fast real-time PCR machine (Applied Biosystems) using standard protocols as recommended by the manufacturer.

### Gait, Balance, and Cognitive Assessments

Inpatients underwent therapist-led examinations of the 10 m walking test, Tinetti gait and balance examination, and MMSE assessments, as per our protocol for the NPH programme (13). After CSF drainage, patients and/or caregivers were asked to grade their own perceived levels of improvement or deterioration at home to the nearest 10%, from—to +100% levels, with 0 being no perceivable difference. A positive response to CSF drainage was defined as an increase of ≥10% in any measure of inpatient gait, balance or cognitive testing (24) or ≥20% functional improvement on the patient's own self-report measure.

### Shunt Implantation

Thirty two patients underwent programmable VPS insertion between 2014 and 2018. Programmable shunt valves (with and without antisiphon devices) were implanted. Shunt valve models were decided according to surgeon's preference; predominantly, Strata (Medtronic) and proGAV (Miethke) valves were used, following all manufacturers' recommendations. Post-operative imaging, comprising a CT brain and shunt series radiographs, was performed to confirm satisfactory placement of all shunt components (proximal to distal). Shunt valve setting adjustments were performed to optimize desired settings to match best patient functional performance. Patients underwent physiotherapy-led clinical assessments and outcomes were recorded using published NPH grading scales. All patients were followed up in outpatient clinic for at least 2 years. In addition, confirmational report was obtained from the patients or their caregivers on any improvement or deterioration in the clinical symptoms i.e., gait disturbance, cognitive impairment, and urinary incontinence.

### Statistical Methods

Statistical analyses were performed using SPSS Statistics Version 23.0 (IBM Corp., Armonk, NY, United States). Continuous variables were analyzed with *t*-tests. Categorical variables were analyzed with the chi-square test. All statistical tests were two-tailed, and significance level was set at 0.05.

## Results

### Patient Characteristics

#### Pilot Study

We have previously published patient characteristics from this pilot cohort elsewhere (13). Following the exclusion as described above, the pilot study cohort comprised of 12 participants (10 male, 2 female) with mean age 71.3 years. All patients presented with gait disturbance and just over half also had cognitive impairment or urinary incontinence/ bladder dysfunction (Table 1). All participants underwent pre-drainage MMSE testing for a baseline assessment, and MMSE scores were not significantly different between responder and non-responder groups.

**Table 1:** Demographic and clinical data for the pilot study.

	**Total**	**Non-responders**	**Responders**
*N*	12	8	4
Age (years)	71.3 ± 7.61	72.8 ± 8.65	68.5 ± 4.66
Sex (male)	10 (0.83)	7 (0.88)	3 (0.75)
**Comorbidity**			
PD or parkinsonism	5 (0.42)	4 (0.50)	1 (0.25)
AD or other dementia	3 (0.25)	2 (0.25)	1 (0.25)
Diabetes mellitus	5 (0.42)	2 (0.25)	3 (0.75)
Hypertension	10 (0.83)	7 (0.88)	3 (0.75)
Hyperlipidaemia	8 (0.67)	4 (0.50)	4 (1.0)
Cardiac disease	4 (0.33)	2 (0.25)	2 (0.50)
CVA/TIA	8 (0.67)	5 (0.63)	3 (0.75)
**Comorbidity scores**			
Charlson Comorbidity Index	1.42 ± 1.08	1.38 ± 1.06	1.50 ± 1.29
Modified Cumulative Illness Rating Scale	14.33 ± 4.70	14.25 ± 5.04	14.50 ± 4.65
Kiefer's Comorbidity Index	3.83 ± 2.52	3.63 ± 2.20	4.25 ± 3.40
**Presenting symptoms**			
Gait disturbance	12 (1.0)	8 (1.0)	4 (1.0)
ADL-Dependent	5 (0.42)	3 (0.38)	2 (0.50)
Tinetti score	15.7 ± 6.99	14.6 ± 7.63	18.3 ± 5.51
Cognitive impairment	7 (0.58)	5 (0.63)	2 (0.50)
MMSE score	20.9 ± 7.12	19.8 ± 7.34	23.3 ± 6.99
Urinary incontinence	7 (0.58)	3 (0.38)	4 (1.0)

*Age and assessment scores presented as mean ± standard deviation; all other variables presented as number of patients (ratio). AD, Alzheimer's Disease; ADL, Activities of Daily Living; PD, Parkinson's Disease*.

10 of 12 patients completed the Tinetti assessment at baseline, with a mean score of 15.7 ± 6.99. 8 of 12 patients were able to complete a 10 m walking test at 0-, 48- , and 72-h CSF drainage. one patient missed their 48-h assessment and responsiveness was based on the assessment at 72 h only. The remaining three patients were not able to undergo either baseline or any gait assessments and their responsiveness was based on other domains tested. Median time for the participant group to complete a 10 m walk was 12.9 Sec (IQR = 11.8–28.5 Sec) at 0 h and 14.8 Sec (IQR = 12.8–19.1 Sec) at 72 h post-CSF drainage.

#### Retrospective Study

Thirty two patients with NPH were included in this study−13 Complex NPH with overlay from neurodegenerative diseases and 19 Classic NPH without overlay from neurodegenerative or significant vascular risks. Among the 13 Complex NPH patients, 6 patients had parkinsonism or Parkinson's disease, 4 patients had Alzheimer's disease, 2 patients had vascular dementia, and 1 patient had mixed Alzheimer's and vascular dementia. There was no significant difference in the age and sex of Complex (73.1 ± 8.62 years; 85% male) and Classic NPH (67.9 ± 7.80 years; 53% male) groups.

All patients presented with gait and balance issues, with mean Tinetti score of 14.2 ± 6.12 in Complex NPH patients and 16.9 ± 8.97 in Classic NPH patients. 77% of Complex NPH and 79% of Classic NPH had cognitive impairment, with mean MMSE score of 20.2 ± 6.61 and 21.6 ± 8.37 respectively. 69% of Complex NPH and 47% of Classic NPH reported urinary symptoms. Although differences in symptom presentation between the groups were not statistically significant, 8 of 13 patients (62%) in the Complex NPH group presented with the complete triad of gait disturbance, cognitive impairment, and urinary incontinence, as compared to 7 of 19 patients (37%) in the Classic NPH group.

### Comorbidities

#### Pilot Study

Main comorbidities present were in the domains of hypertension (83%), neurological (83%), endocrine/metabolic (75%), genitourinary (58%), musculoskeletal (42%), and cardiac (33%). 42% had a diagnosis of parkinsonism. We were unable to distinguish vascular risk characteristics unique to responders. Mean scores were 3.83 ± 2.52 using the CMI, 1.42 ± 1.08 using the CCI, and 14.3 ± 4.70 using the modified CIRS. No significant differences were observed between responders and non-responders on all comorbidity scales, although the responders tended toward higher comorbidity scores overall.

#### Retrospective Study

**Figure 2:**
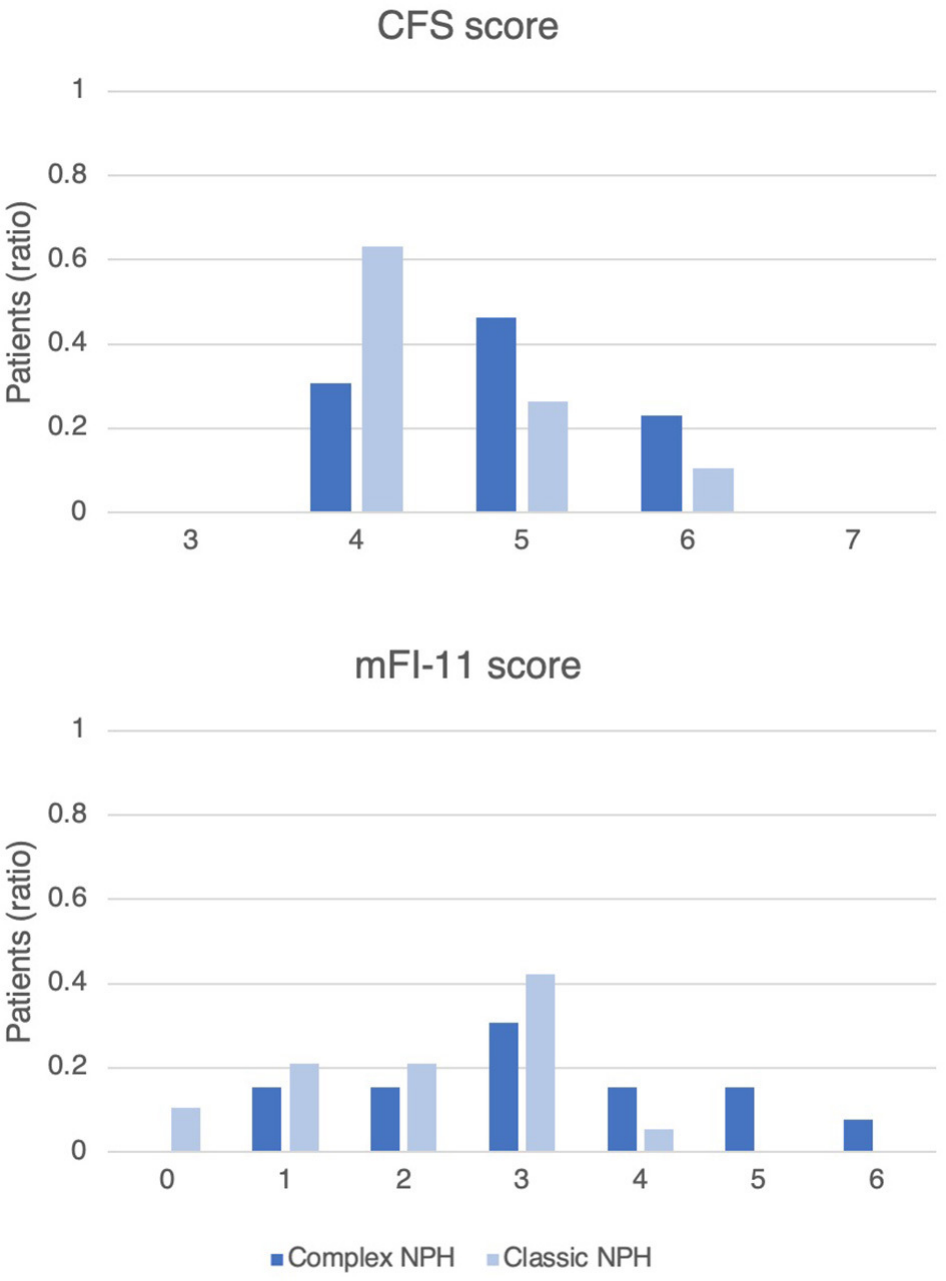
Histogram of frailty scores on the CFS and mFI-11, demonstrating the distribution of frailty risk in Complex NPH and Classic NPH patients. The y-axis represents the ratio of patients in each group.

The retrospective cohort mainly had comorbidities of hypertension (78%), hyperlipidaemia (59%), and diabetes (41%). The proportion of patients with comorbid conditions was not significantly different in Complex NPH and Classic NPH patient cohorts (Table 2). For frailty risk, Complex NPH patients had significantly higher scores on the mFI-11, 3.23 ± 1.54 compared to 2.11 ± 1.15 for Classic NPH patients. Figure 2 demonstrates the distribution of frailty scores in the two groups.

**Table 2:** Demographic and clinical data for the retrospective study.

	**Total**	**Complex NPH**	**Classic NPH**	***P***
*N*	32	13	19	
Age (years)	70.0 ± 8.41	73.1 ± 8.62	67.9 ± 7.80	0.087
Sex (male)	21 (0.66)	11 (0.85)	10 (0.53)	0.061
**Comorbidity**				
PD or parkinsonism	6 (0.19)	6 (0.46)	0 (0.0)	0.001
AD or other dementia	7 (0.22)	7 (0.54)	0 (0.0)	<0.001
Diabetes mellitus	13 (0.41)	5 (0.38)	8 (0.42)	0.837
Hypertension	25 (0.78)	8 (0.62)	17 (0.89)	0.060
Hyperlipidaemia	19 (0.59)	6 (0.46)	13 (0.68)	0.208
Cardiac disease	9 (0.28)	3 (0.23)	6 (0.32)	0.599
Vascular disease	3 (0.09)	1 (0.08)	2 (0.11)	0.787
CVA/TIA	8 (0.25)	4 (0.31)	4 (0.21)	0.533
**Frailty**				
CFS score	4.66 ± 0.75	4.92 ± 0.760	4.47 ± 0.697	0.094
mFI-11 score	2.56 ± 1.41	3.23 ± 1.54	2.11 ± 1.15	0.024
**Presenting symptoms**				
Gait disturbance	32 (1.0)	13 (1.0)	19 (1.0)	N.A.
Tinetti score	15.9 ± 7.99	14.2 ± 6.12	16.9 ± 8.97	0.354
Cognitive impairment	25 (0.78)	10 (0.77)	15 (0.79)	0.892
MMSE score	21.0 ± 7.57	20.2 ± 6.61	21.6 ± 8.37	0.601
Urinary incontinence	18 (0.56)	9 (0.69)	9 (0.47)	0.221
**Improvement after VPS**				
Gait disturbance	28 (0.88)	13 (1.0)	15 (0.79)	0.077
Post-shunt Tinetti score	20.2 ± 6.66	18.4 ± 5.76	21.4 ± 7.11	0.238
Cognitive impairment	17 (0.53)	3 (0.23)	14 (0.74)	0.005
Urinary incontinence	8 (0.25)	3 (0.23)	5 (0.26)	0.835

*Age and assessment scores presented as mean ± standard deviation; all other variables presented as number of patients (ratio). AD, Alzheimer's Disease; CFS, Clinical Frailty Scale; CVA, cerebrovascular accident; mFI-11, 11-factor modified frailty index; NPH, Normal Pressure Hydrocephalus; PD, Parkinson's Disease; TIA, transient ischaemic attack*.

### Correlation Between Imaging Biomarkers, *APOE* Genotyping and CSF Responsiveness

**Figure 3:**
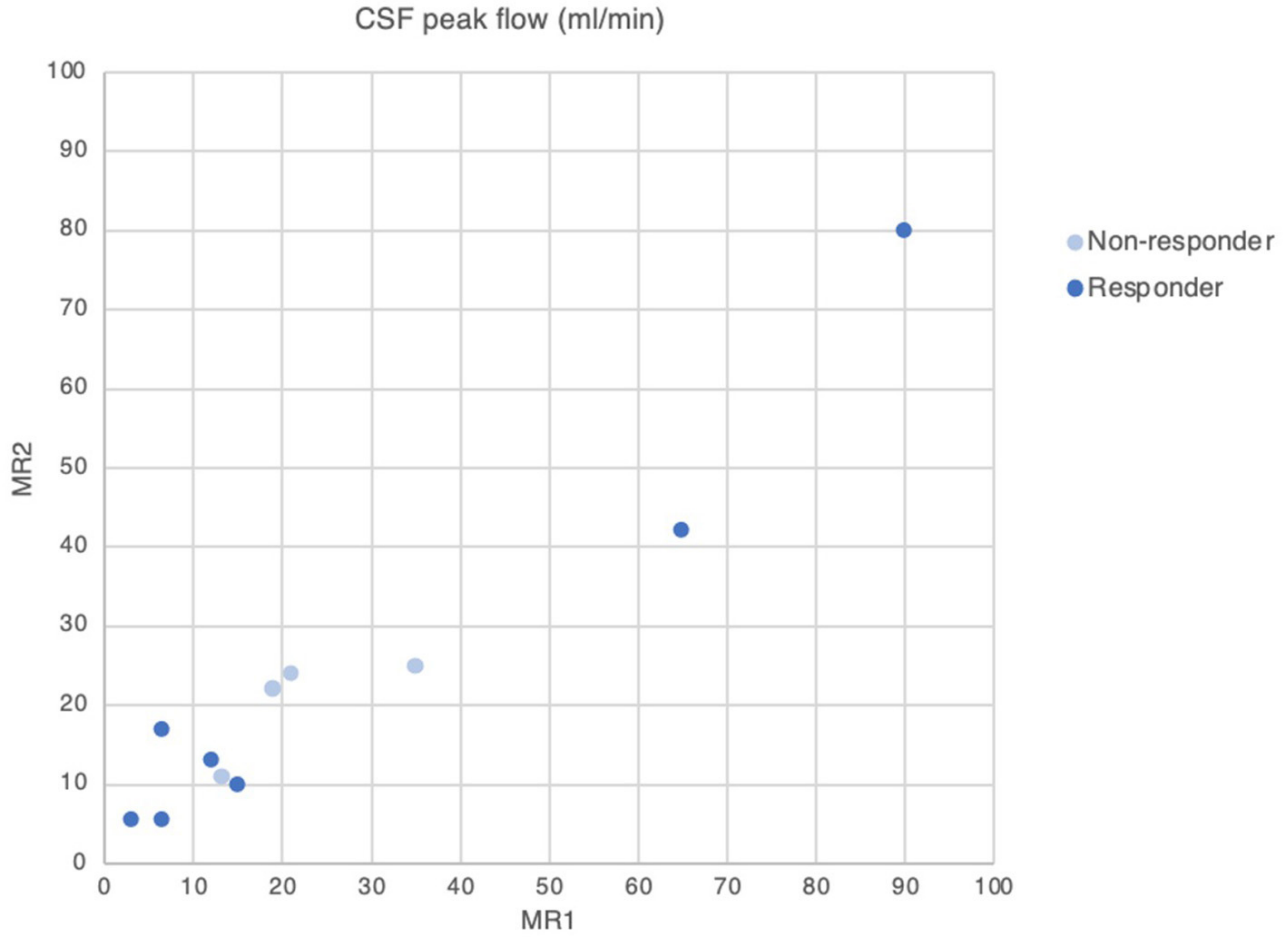
CSF peak flow in NPH patients before (MR1) and after (MR2) CSF drainage.

All patients exhibited ventriculomegaly with Evans' and Bicaudate indices ≥0.30 and ≥0.25 respectively. The degree of ventriculomegaly did not distinguish between responders vs. non-responders; there were no significant differences post-drainage in either group. CSF drainage did not influence CSF peak flow rates consistently in either group. One patient was excluded from CSF flow analysis for technical reasons. However, CSF peak flow values showed an interesting dichotomy between groups. Non-responders demonstrated both low and ultra-high values at baseline (3–15 and 65–90 ml/min), whereas responders only demonstrated CSF peak flow in a clustered range (13.2–35 ml/min—Figure 3).

White matter hyperintensities scored by the Fazekas scale did not distinguish between responder and non-responder groups.

*APOE* genotyping for 11 participants did not demonstrate a difference between responders and non-responders in genetic predisposition toward developing Alzheimer's disease (Table 3). One non-responder was excluded due to inadequate sample. As the sample size was small, no statistical analysis was performed to compare the distribution of the *APOE* genotypes between groups. As expected, the ε3ε3 genotype was the most common across both groups; this is the most common genotype universally. The ε3ε4 genotype carrying the ε4 risk allele was detected in both groups (1 in 4 responders vs. 1 in 7 non-responders), whilst the protective ε2 allele was found in a responder with the ε2/ε3 genotype (1 in 4 responders).

**Table 3:** *APOE* genotypes in the pilot study cohort.

***APOE* genotype**	**Non-responders** **(*n* = 7)**	**Responders** **(*n* = 4)**
ε2/ε2	0	0
ε2/ε3	0	1
ε2/ε4	0	0
ε3/ε3	6	2
ε3/ε4	1	1
ε4/ε4	0	0

*APOE ε4 is the main genetic risk factor for Alzheimer's disease. The risk associated ε4 allele conveys an increase in risk of around 2–3x in heterozygous form, and around 15x in homozygous form, while the ε2 allele is associated with decreased risk*.

### Patient Outcomes

#### Pilot Study

We have previously reported clinical outcomes for this pilot study cohort of patients with Complex NPH (13). In summary, there were four CSF responders (and one patient subsequently excluded from analysis due to lack of compliance) who were offered surgical intervention. Six ventriculo-peritoneal shunts were inserted; one procedure was a shunt reinsertion for a patient who required shunt externalization due to a non-infected pseudocyst. Two responders demonstrated higher than expected levels of improvement. Of the non-responders, one patient died from a cerebrovascular accident and another improved post-discharge but declined intervention.

#### Retrospective Study

Post-surgery, three patients had infections requiring shunt removal, and one patient underwent a shunt revision due to blockage of the shunt. Two patients passed away within 3 years of shunt insertion; one developed sepsis secondary to ventriculitis, and the other had a subdural haematoma after a fall (Table 4).

**Table 4:** Outcomes and complications for the retrospective study.

	**Complex NPH**	**Classic NPH**
Shunt revision	0 (0.0)	1 (0.05)
Shunt infection requiring shunt removal	1 (0.08)	2 (0.11)
Deterioration after initial improvement	2 (0.15)	1 (0.05)
Passed away within 3 years of shunt insertion	1 (0.08)	1 (0.05)

*Data presented as number of patients (ratio)*.

After VPS insertion, significantly more Classic NPH patients had improved cognition compared to the Complex NPH group (*p* = 0.005). Improvement in gait and urinary symptoms was not significantly different between the groups. 26% of the Classic NPH group showed global improvement of the triad, and 42% improved in two domains. Although only 8% showed global improvement of the triad, all Complex NPH patients improved in gait. Tinetti scores were significantly higher in both groups after VPS insertion (*p* <0.001 in Complex and *p* = 0.002 in Classic NPH).

## Discussion

Comorbidities and frailty confound the diagnosis of NPH in older people. With the increasing trend toward a rise in the aging population, encountering patients with concurrent NPH and neurodegenerative disease burden is becoming increasingly common. Modern guidelines and best practices are predominantly focused upon idiopathic NPH, almost at the exclusion of patients who have overlay from neurodegenerative diseases and significant comorbidity burden. Highly invasive procedures such as brain biopsy or advanced imaging biomarkers including SPECT imaging or DTI profiles have shown promise in their utility toward more precise characterization and prognostication of such complicated patients (25, 26). However, the usefulness of such tools may be limited by time, expertise, and resources available at the clinical-research interface.

NPH represents a whole spectrum of disease that is greater than the sum of its parts; indeed, it may be considered a model disorder for the development of a new paradigm of clinical thinking, i.e., toward geriatric neurosurgery as a formal sub-specialization of increasing importance. Our study supports such aims. By proposing a more simplistic conceptual framework of characterizing NPH into two distinct cohorts of Classic vs. Complex forms of disease, we aim to aid in a more rapid shorthand for clinical risk stratification and expectations for treatment strategies. We believe ours to be the first study presenting a multimodal biomarker characterization specific to Complex NPH, with a further study documenting longer-term clinical outcomes.

### Characterization of Complex NPH

Our pilot study has demonstrated that, in patients with Complex NPH, the presence of a comorbidity burden does not preclude responsiveness to CSF drainage. We found that risk assessment based on NPH-specific comorbidity indices was insufficient in patients with Complex NPH. In Classic NPH, significant differences have been demonstrated between patients with excellent and poor outcome following shunting using the CMI (2 vs. 4 points, *p* = 0.001) (15). Our data confirm that, in patients with Complex NPH, the CMI did not significantly distinguish between responders and non-responders; there was, in fact, a trend toward a higher comorbidity burden in responders. CMI was also not correlated to gait and Tinetti assessment scores.

Such findings are consistent with observations previously noted in NPH literature on comorbidities (12). The ISHCSF report recommended that global measures of risk burden be considered (such as for cardiovascular or ischaemic stroke conditions) when evaluating risk factor profiles. Our data support that need. We have demonstrated, using the modified CIRS and CCI scores, that mean global comorbidity scores of patients with Complex NPH were better than published scores of their hospitalized elderly peers, for whom a range of interventions are routinely considered. This implies that patients with Complex NPH should not be declined surgical intervention based on comorbidities alone. To our knowledge, this is the first study to use both NPH-specific and global risk stratification scores in Complex NPH.

Such global risk scales also distinguished between comorbidity risks and responses to CSF drainage. Our study revealed that responders and non-responders had differing profiles of comorbidity risks. A higher overall burden of comorbidities significantly correlated with cognitive impairment in the dementing range at presentation. Certain domains appeared predominant within Complex NPH patients, i.e., vascular risk burden (cardiac disease and hypertension domains) and functional symptomatology (genitourinary, psychiatric and behavioral scores). Hypertension and parkinsonism outweighed other comorbidities in predisposing to non-response following extended CSF drainage. Surprisingly, we found a heavy load of individual comorbidity risks in responders. These included dementia (other), cardiac disease and hyperlipidaemia (all twice as common in responders), cerebrovascular disease (13% higher), as well as diabetes (three times as common). We therefore concluded that there was no evidence that individual comorbidity risks directly correlated with lack of response, but some risks appeared more important than others. Overall, as expected, most of the group were non-responders. The response rate in Complex NPH was lower than that expected of Classic NPH (approximately 33% vs. 60–70% responders) (25, 27). However, responses were comparable to in-house rates for extended CSF drainage in Classic NPH patients without significant comorbidities, but borderline responses to lumbar tap testing.

The striking trends of comorbidity burden in responders may be due to the smaller sample of responders, leading to overrepresentation of risks. However, certain patterns merit further discussion. NPH patients are known to have a higher vascular risk burden than expected (12). Hypertension is more common in NPH compared to age-matched controls, even where other neurological disorders are present. This implies a possible causality in the relationship between hypertension and NPH pathophysiology. It is thought that arterial pulsations in the ICP curve may contribute to the development of progressive ventriculomegaly. However, it is currently unknown if, in established NPH, vascular risk factors actively influence the response of CSF drainage and if so, how they should be scored. Further work is required to understand if remediation of vascular risk factors would promote enhancement of either responsiveness of ventriculomegaly to intervention or host response to compensating for hydrocephalus. Despite this, our results suggest that vascular risk burden, and specifically hypertension, does not necessarily preclude a favorable response to extended CSF drainage.

We also found that vascular imaging burden, namely periventricular and deep white matter hyperintensities, did not distinguish between responders and non-responders. White matter lesions did not improve following CSF drainage in either group. Whilst it could be argued such lesions should be permanent, other groups have demonstrated changes in white matter hyperintensities following CSF drainage in NPH (26). Equally, we found no significant differences in structural measurements for ventriculomegaly, such as Evans' and Bicaudate indices, pre- and post-CSF drainage. This suggests that static uniplanar measurements for ventriculomegaly may be unhelpful in determining the degree of responsiveness in Complex NPH. CSF flow measurements are a more dynamic method of interrogating CSF disturbance but are subject to a multitude of technical considerations (28).

We found that, in Complex NPH, CSF peak flow rates were dichotomized according to group. Non-responders demonstrated CSF peak flow rates at both extremes of low and ultra-high values. By contrast, CSF peak flow rates for responders clustered near the thresholds of 18–24.5 ml/min found in classic shunt-responsive NPH (29, 30). CSF flow dynamics from our Complex NPH cohort, who present late for intervention due to investigations for comorbidities, appear to confirm findings from CSF infusion studies that resistance to CSF outflow have a strong tendency to decrease with time with the duration of symptoms beyond 2 years (31). This suggests there exists a threshold, beyond which damage from CSF disturbance becomes irreversible, leading to likely shunt failure even in the presence of radio-physiological biomarkers of shunt-responsiveness. Recent work from our group confirms the presence of such cohorts (32).

### The Value of a Classic vs. Complex NPH Framework

Our retrospective study focused on comparing patients with Classic NPH vs. Complex NPH; the latter group comprise patients presenting with features of NPH, as well as a spectrum of neurodegenerative diseases including parkinsonism/Parkinson's disease, Alzheimer's disease, and vascular dementia. The patients in the group termed Complex NPH were older than the group with Classic NPH. This finding is consistent with current published literature (2, 3, 8, 9). There was no significant difference in distribution of sex in the published work whilst our data showed a predominance in males (8, 9). However, this is likely to reflect the small sample size of our study.

It is not surprising that the group without neurodegenerative disorders did better. However, it was striking that, despite older age and overlay from neurodegenerative disease, all patients with Complex NPH showed improvement in their gait. However, only 15% in this group showed improvement in cognition or urinary incontinence. The published literature regarding coexistence of Alzheimer's disease and NPH are few and their conclusions vary. Savolainen et al. postulated that the relatively high prevalence of Alzheimer's disease in patients with NPH may explain the unsuccessful recovery of many patients after shunt placement and hence recommended cortical brain biopsy (6). Conversely, studies by Golomb et al. and Yasar et al. concluded that concomitant Alzheimer's disease pathology does not strongly influence the clinical response to shunt surgery (7, 9). A published study in 2015 by Pomeraniec et al. found that those NPH patients with AD will ultimately suffer recurrence and worsening of presenting symptoms, though majority of these patients enjoyed initial clinical improvement following ventriculo-peritoneal shunting (8).

There is a paucity of published data regarding specific outcomes from the concurrence of NPH and vascular dementia; this is likely because the relationship between small vessel diseases to the pathogenesis of NPH has been postulated (28, 33). Conversely, more work has been published concerning NPH with Parkinsonism and its correlation with dopaminergic degeneration. Some studies proposed Parkinsonism to be assessed as one of the outcome measures—these studies had reported good clinical responses after shunt surgery. A recent study done in Taiwan advised using ^99m^Tc- TRODAT-1 SPECT scans to look for dopaminergic degeneration in order to predict the surgical outcomes because this comorbidity was thought to neutralize the degree of improvement after surgery (12).

Due to the heterogeneity of neurodegenerative disorders, their presentations from typical to atypical and differences in clinical diagnostic criteria, it may be argued that the presence of neurodegenerative diseases themselves may confound the understanding of Complex NPH *per se*. It may be that some neurodegenerative diseases are more important to the understanding of NPH compared to others.

Indeed, some authors have suggested that NPH and Alzheimer's disease are closely connected and may simply occupy different extremes of a common spectrum of disease (34). The deposition of amyloid plaques, the hallmark of Alzheimer's disease, has been proven in NPH, in both PET and cortical biopsy studies (35, 36). A heavier burden of amyloid deposition is thought to predispose to non-responsiveness to shunting. We expected to confirm the presence of risk factors for neurodegenerative disease within the complex NPH cohort. However, similar to findings in classic NPH (37), we found the majority of Complex NPH patients lacked the genotypic risk factor for late-onset Alzheimer's disease. The *APOE* ε4 allele was equally demonstrated in both responder and non-responder groups (*n* = 1 each), although the *APOE* ε2 allele phenotype, thought to be a protective factor, was only exhibited in one responder. The presence of this protective allele in NPH and its contribution to CSF responsiveness is largely unknown in literature but we note our small sample size is a limitation in our further interpretation of these results.

The myriad of neurodegenerative disease subtypes and presentations demonstrates the value of a more simplistic framework of NPH distinguishing Classic vs. Complex NPH cohorts. This is because the specific diagnostic criteria, or lack thereof, of neurodegenerative disorder is not as critical to CSF responsiveness as quantifying the “NPH component of disease” remediable to surgical intervention, regardless of such overlay. We apply the same rationale to the inclusion of significant comorbidities as criteria for Complex NPH; specifically scoring individual comorbidity and frailty burdens alone did not distinguish the two NPH cohorts, whereas regarding their clinical risk stratification as a whole was more effective in separating their differing thresholds of outcomes. In more recent work, we have suggested a further refinement of the term Complex NPH, by expanding on our definition for consistency (38), to address shortcomings in our preliminary approach to this concept.

### Limitations

Our study had several limitations. Firstly, our pilot study focused upon the assessment of global comorbidity risks in patients with complex NPH undergoing extended CSF drainage, without considering the wider implications of such findings either to longer-term shunting or the pathophysiology of NPH itself. Further work is needed to characterize the contribution of specific components of vascular risk burden (for example, cardiac vs. hypertensive risks), as well as individual neurodegenerative diseases, such as Alzheimer's and Parkinson's diseases. Functional outcome is difficult to test in such patients. The group size was small and reflects both the relatively low incidence of NPH, as well as the smaller subgroup of complex NPH within the overall spectrum of disease.

Despite this, our study numbers do match previous published cohort sizes for extended lumbar drainage (26). Due to the small group size, however, comorbidities and other risks may appear to be overrepresented within the groupings, especially within the responders. This is also true of complication rates, which may appear higher due to the small sample size (16.7% in 6 shunts). Interestingly, these are similar thresholds to the 12–13% complication rates reported by recent large international series in classic NPH (15, 39) and lower than others (40). It may also be possible to pursue strategies to achieve complication rates <10% with modern shunting (27, 41). Although the response rate in Complex NPH with comorbidities is lower than in NPH without major comorbidities, the risk-benefit ratio for such patients still favors intervention if complication rates for shunting remain comparable to that of Classic NPH.

Indeed, our larger retrospective study demonstrated much lower complication rates specific to shunting interventions, although decline and mortality from overlay of other conditions were still important determinants of longer-term outcomes. In addition, as described above, our aim was to develop a more simplistic conceptual framework for rapidly screening NPH subtypes according to expectations of treatment outcomes. More precise tools for confirming and characterizing neurodegenerative disorders, such as the use of brain biopsy, CSF biomarkers and advanced imaging tools, such as SPECT and DTI, may offer more sophisticated ways of distinguishing these cohorts.

## Conclusions

Our study has demonstrated that the presence of neurodegenerative disorders co-existing with NPH should not be the sole barrier to the consideration of high-volume tap test or lumbar drainage via a specialist NPH programme. Further characterization of distinct cohorts of NPH with differing degrees of CSF responsiveness due to overlay from neurodegenerative or comorbidity risk burden may aid toward more precise prognostication and treatment strategies. We propose a simplistic conceptual framework to describe NPH by its Classic vs. Complex subtypes to promote the clinical paradigm shift toward subspecialist geriatric neurosurgery by addressing needs for rapid screening tools at the clinical-research interface.

## Data Availability Statement

The raw data supporting the conclusions of this article will be made available by the authors, without undue reservation.

## Ethics Statement

The studies involving human participants were reviewed and approved by the SingHealth Centralised Institutional Review Board. Written informed consent was obtained from participants of the pilot study. The ethics committee waived the requirement of written informed consent for participation in the retrospective study.

## Author Contributions

NK, CL, and ETG contributed to the conceptualization and design of the study and wrote the manuscript. ETG, CL, AT, BLT, SL, and JK contributed to data collection and analysis. SK contributed to design of the imaging protocol as well as interpretation. AA–A and VN contributed to genomic analysis. RP, YJT, AN, EKT, ZC, MC, and JP contributed to the characterization of the NPH cohorts and provided feedback on the project. All authors contributed to the article and approved the submitted version.

## Funding

This study was supported by grants from the National Neuroscience Institute Center Grant (NCG CS03) and the National Medical Research Council (NMRC/TA/0024/2013 and MOH-CSAINV18nov-0005).

## Conflict of Interest

The authors declare that the research was conducted in the absence of any commercial or financial relationships that could be construed as a potential conflict of interest.

## Publisher's Note

All claims expressed in this article are solely those of the authors and do not necessarily represent those of their affiliated organizations, or those of the publisher, the editors and the reviewers. Any product that may be evaluated in this article, or claim that may be made by its manufacturer, is not guaranteed or endorsed by the publisher.
